# Monocyte chemoattractant protein-1 as a potential marker for patients with sepsis: a systematic review and meta-analysis

**DOI:** 10.3389/fmed.2023.1217784

**Published:** 2023-09-01

**Authors:** Zhuo Chen, Chenwei Li, Jian Yu

**Affiliations:** ^1^Department of Intensive Care Unit, The Second Hospital of Dalian Medical University, Dalian, China; ^2^Department of Pulmonary and Critical Care Medicine, The Second Hospital of Dalian Medical University, Dalian, China

**Keywords:** diagnosis, meta-analysis, MCP-1, sepsis, biomarker

## Abstract

**Objective:**

To investigate the diagnostic value of monocyte chemoattractant protein-1 (MCP-1) as a biomarker for adult patients with sepsis.

**Methods:**

Related studies on the diagnostic value of MCP-1 in adult patients with sepsis were searched in PubMed, Cochrane Library, Embase, CNKI, CBM, Web of Science, Scopus, and Wanfang Data databases (published to February 20, 2023) was performed if studies assessed the diagnostic accuracy of MCP-1 in adult patients with sepsis and provided appropriate information sufficient to construct a 2 × 2 linked table, studies were included.

**Results:**

Data from 8 studies with a total of 805 patients were included. The combined sensitivity was 0.84 (95% CI 0.70–0.92), the specificity was 0.82 (95% CI 0.67–0.91), the combined positive likelihood ratio was 3.711 (2.119–6.500), the negative likelihood ratio was 0.287 (0.198–0.415), and the area under the working characteristic curve for combined subjects was 0.88. The diagnostic odds ratio (DOR) was 16.508 (7.632–35.706). Meta-regression analysis showed that the results were not significant. Deeks’ funnel plot showed that there was no publication bias.

**Conclusion:**

According to our meta-analysis, MCP-1 is a valuable biomarker and may provide evidence for the diagnosis of sepsis in adults.

## 1. Introduction

Sepsis is defined as life-threatening organ dysfunction caused by a dysregulated host response to infection. Over 30 million patients were diagnosed with sepsis worldwidely each year with a mortality of 15%–25% (1). The inflammatory cytokine storm caused by excessive activation of immune system is crucial in treatment of sepsis, which is the main reason of the rapid progression and multiple organ dysfunction (2). Early diagnosis of sepsis is important for early goal-directed bundle therapy, improving treatment capabilities, and prognosis (3). It is still difficult to distinguish sepsis from non-bacterial systemic inflammation response syndrome (SIRS), such as surgery, trauma, and pancreatitis without infection. Therefore, finding sepsis-related biomarkers for early diagnosis has become one of the research directions.

As early as the 1980s, a series of studies showed that the plasma level of proinflammation cytokine including tumor necrosis factor-α (TNF-α), interleukin-1β (IL-1β), and interleukin-6 (IL-6) were related to the severity and prognosis of sepsis. Elevated levels of IL-6 and IL-8 can predict the occurrence of neonatal sepsis (4). In 2003, C-reactive protein (CRP) and procalcitonin (PCT) were also included in the sepsis-2 guidelines (5); however, CRP has low specificity and hysteresis (6). The plasma level of PCT usually rises after 4 h of infection and comes to the peak after 8–24 h, which is too late for the 6 h bundle therapy for sepsis treatment. Currently, the primary basis for diagnosing sepsis is the Sequential Organ Failure Assessment (SOFA) score containing many indicators and has the same hysteresis in detection (7). Therefore, finding and applying biomarkers for sepsis diagnosis with high sensitivity and specificity in clinical practice is urgent.

Monocyte chemoattractant protein-1 (MCP-1), also known as chemokine (CC-motif) ligand 2 (CCL2), is a member of the CC chemokine family that interacts with the chemokine receptor-2 (CCR2) on the cell surface and promotes the expression of other inflammatory factors/cells. It mediates the migration and infiltration mechanism of inflammatory cells (such as monocytes/macrophages and other cytokines) at the site of inflammation. It has been figured out that it is involved in the SIRS and tumor occurrence (8). Multiple studies have shown that MCP-1 level is significantly elevated in sepsis patients and it is also associated with multiple organ dysfunction syndrome (MODS) (9). It may be a more “direct” marker of infection (10).

To better clarify the value of MCP-1 in diagnosing sepsis, this study conducted a systematic review and meta-analysis of published MCP-1-related literature, then integrated and evaluated its application value in sepsis diagnosis.

## 2. Materials and methods

This study was performed in compliance with the international platform of registered systematic review and meta-analysis protocols and registered as CRD42023409147 on PRESPERO.

### 2.1. Search strategy

We systematically searched PubMed, Cochrane Library, Embase, China National Knowledge Infrastructure (CNKI), Chinese Biomedical Literature Database (CBM), Web of Science, Scopus, and Wanfang Data for literature containing MCP-1 detection for sepsis diagnosis. The research was searched in Chinese and English databases using the subject words and free words of “sepsis,” “bloodstream infection,” “monocyte chemoattractant factor-1,” “MCP-1,” and “predictive,” combined with MeSH subject terms, from the establishment of the database to February 20, 2023. Language restrictions were set to Chinese and English. Relevant materials and conference papers in Chinese and English were manually searched, and the attached references were consulted. A detailed search strategy is published in the Supplementary material.

### 2.2. Inclusion and exclusion criteria

#### 2.2.1. Inclusion criteria

(1) Study design: the clinical studies of MCP-1 in the diagnosis of sepsis, including prospective studies and retrospective studies, are limited to Chinese and English. (2) Subjects: the case group was adult hospitalized patients with confirmed sepsis. (3) Diagnostic test to be evaluated: plasma concentration of MCP-1 was measured. (4) True positive values (TP), false positive values (FP), false negative values (FN), and true negative values (TN) for MCP-1 diagnosis of sepsis can be directly extracted or indirectly calculated. (5) Diagnostic criteria: sepsis was diagnosed according to the “Third International Consensus Definition for Sepsis and Septic Shock” guideline in 2016 (1).

#### 2.2.2. Exclusion criteria

(1) Non-clinical diagnostic studies. (2) Literature that cannot extract data from the four-grid table or has obvious errors in the original research. (3) Animal experiments, dissertations, literature reviews, case reports, etc.

### 2.3. Literature screening and data extraction

Two researchers independently screened the literature and extracted data. When there were differences in the screening results, a third person re-evaluated them, and the results were discussed among them. The data information extracted in the study includes: (1) general information: title, first author, publication time; (2) study characteristics: diagnostic gold standard, detection method, cut-off point, study subjects (number, age, source of cases); (3) diagnostic threshold, an area under the curve (AUC), and TP, FP, FN, TN values are directly or indirectly extracted, and summarized into tables. The quality of the literature was evaluated using the QUADAS-2 criteria in Revman 5.4 software (11).

### 2.4. Quality assessment

The QUADAS-2 tool in Review Manager 5.4 was used to evaluate the quality of the studies. The four aspects of case selection, trials to be evaluated, gold standard, case flow, and clinical applicability were judged as “yes,” “no,” or “unclear,” and the corresponding risk levels were defined as “low risk,” “high risk,” and “uncertain,” i.e., when the answer for a particular item is “yes,” the risk of bias is considered low. If the answer is “no,” the possibility of bias is considered, and if the answer is “unclear,” there is not enough data to judge the risk of bias.

### 2.5. Statistical analysis

MetaDisc V1.4 and STATA 16.0 software were used to analyze and merge sensitivity, specificity, and their corresponding 95% confidence intervals, combined positive likelihood ratio (PLR), negative likelihood ratio (NLR), and diagnostic odds ratio (DOR), and use mixed frequency data sampling regression models as installed in STATA 16 to draw a summary receiver operating characteristic (sROC) curve. The AUC and Q* index were calculated to determine the diagnostic value of MCP-1 for sepsis. Spearman’s coefficient was used to determine whether the threshold effect caused heterogeneity, and the Cochrane-*Q* value of DOR was calculated to test for non-threshold effects. The *I*^2^ statistic was used to test for heterogeneity in the included studies. *I*^2^ < 50% indicates non-significant heterogeneity and a fixed-effect model is used. When *I*^2^ > 50%, significant heterogeneity is present, a random-effect model is used, and subgroup analysis is performed to determine the source of heterogeneity. A sensitivity analysis was performed to evaluate the stability of the results. Publication bias was assessed using Deeks’ funnel plot (12).

## 3. Result

### 3.1. Literature search and study characteristics

**Figure 1:**
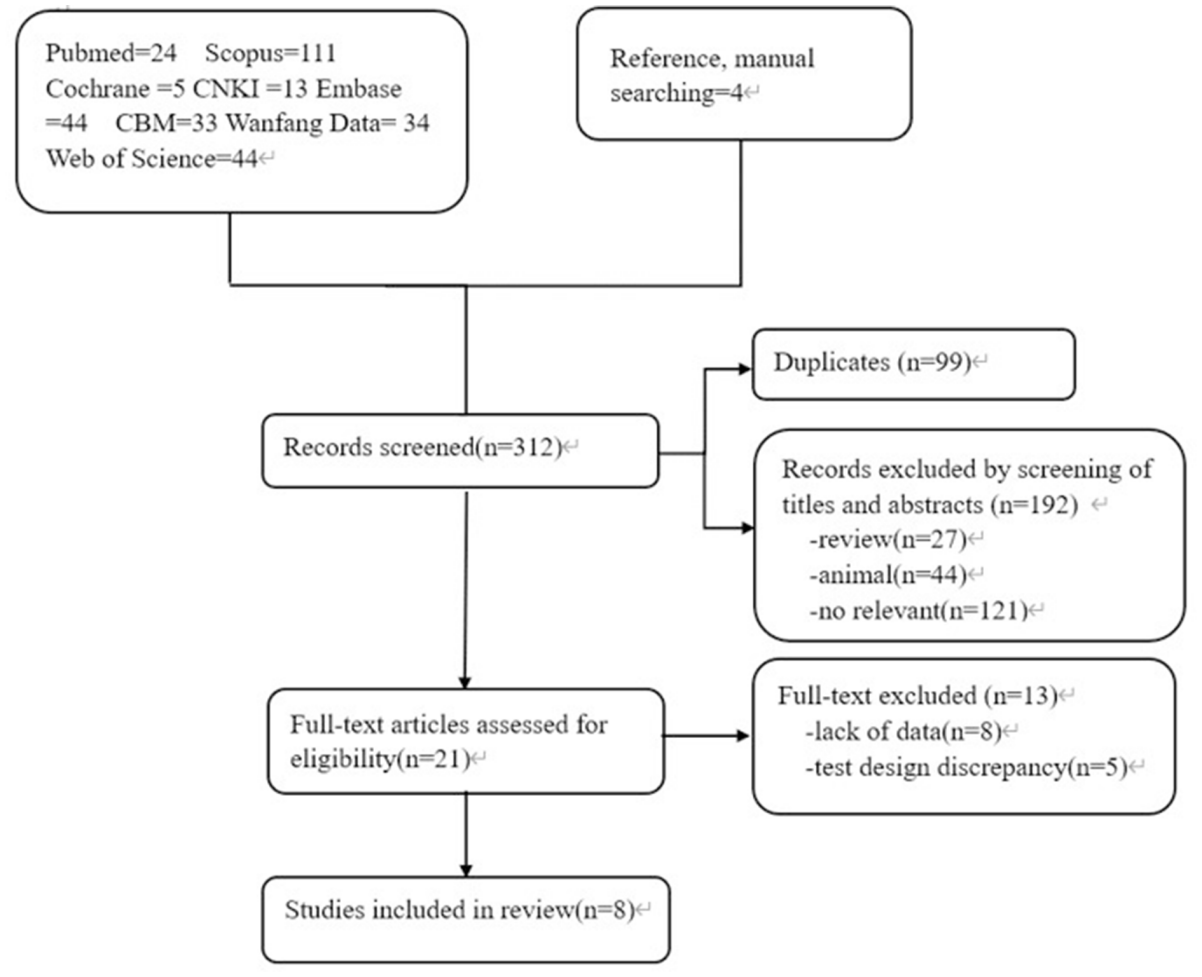
Flow chart of literature.

A total of 312 articles were collected, including 78 Chinese articles and 234 English articles. After excluding 99 duplicate articles, 71 animal experiments/literature reviews/conference reports, and 121 articles with content that did not meet the research criteria after reading the abstract, 8 studies (13–20) were finally included after reading the full text and referring to the references. The flowchart of literature screening is shown in Figure 1. A total of 802 patients were included, including 449 patients with sepsis or septic shock and 353 non-sepsis patients. Five studies included patients from the Intensive Care Unit (ICU) (14, 16–18, 20). Only one study (14) used quick-SOFA (qSOFA) as the diagnostic criterion for sepsis, and the other 7 studies used suspected infection + SOFA ≥2 points as the criterion for sepsis. Enzyme linked immunosorbent assay (ELISA) was used as the detection method in 5 studies (13, 14, 17, 19, 20), and different brands of multi-factor integration collection and detection methods were used in the other 3 studies (15, 16, 18). The basic clinical characteristics of the included studies are shown in Table 1.

**Table 1:** Basic characteristics of included studies.

Author	Year	Region	Size	Source department	Male	Test method	Sensitive	Specificity	TP	PF	FN	TN	Cut-point (pg/mL)	AUC
Duman	2018	Turkey	88	ICU	60.0%	ELISA	97.80%	73.80%	45	11	1	31	125.19	0.808
Gao	2023	China	120	ED	53.8%	Quantibody^®^Human Inflammation Array 1	74.76%	74.95%	60	10	20	30	NA	0.765
Jekarl	2019	Korea	160	ICU	55.0%	45-Plex Human ProcartaPlex Panel 1	67.50%	93.00%	54	6	26	74	105.5	0.84
Wang	2018	China	72	Traumatic department	71.8%	ELISA	92.86%	43.75%	36	19	3	14	240.7	0.82
Xu	2022	China	80	RICU	52.5%	ELISA	97.50%	100.00%	39	0	1	40	14.28	0.996
Li	2022	China	168	NA	50.6%	ELISA	75.00%	85.71%	84	8	28	48	20	0.825
Pyle	2011	America	63	ICU	NA	ELISA	65.00%	76.00%	17	9	9	28	642	0.7
Tian	2019	China	51	ICU	62.7%	Luminex Multiplex Assay	69.23%	70.59%	18	7	8	18	382	0.716

### 3.2. Literature quality evaluation

**Figure 2:**
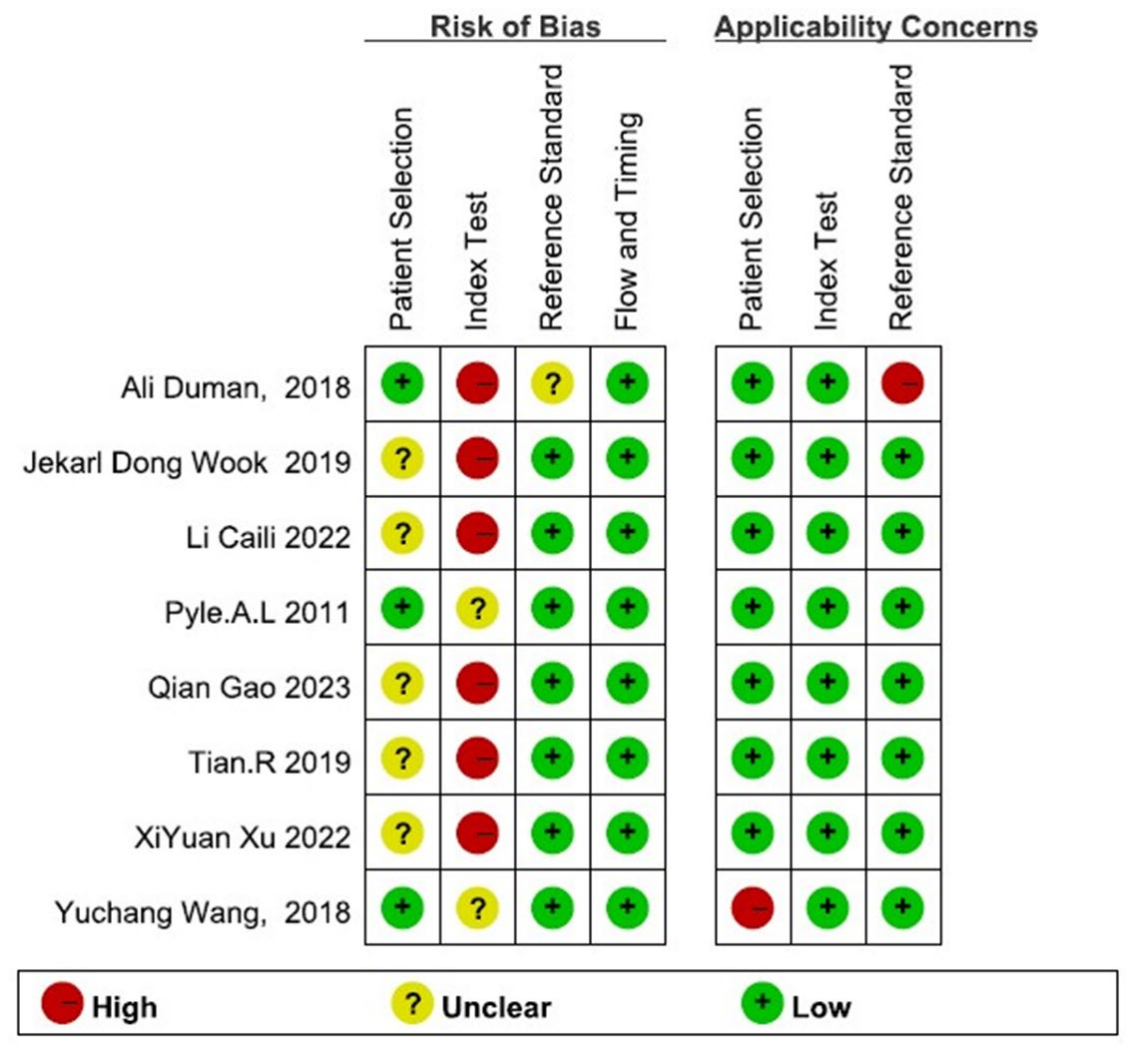
Risk of bias and applicability concerns of the included studies. The overall results showed that the bias risk of the included studies was not high, and the bias had little impact on the analysis results.

The 8 studies included generally needed more detailed descriptions of the case selection process. However, the overall results showed that the bias risk of the included studies was not high, and the bias had little impact on the analysis results (Figure 2).

### 3.3. Publication bias

**Figure 3:**
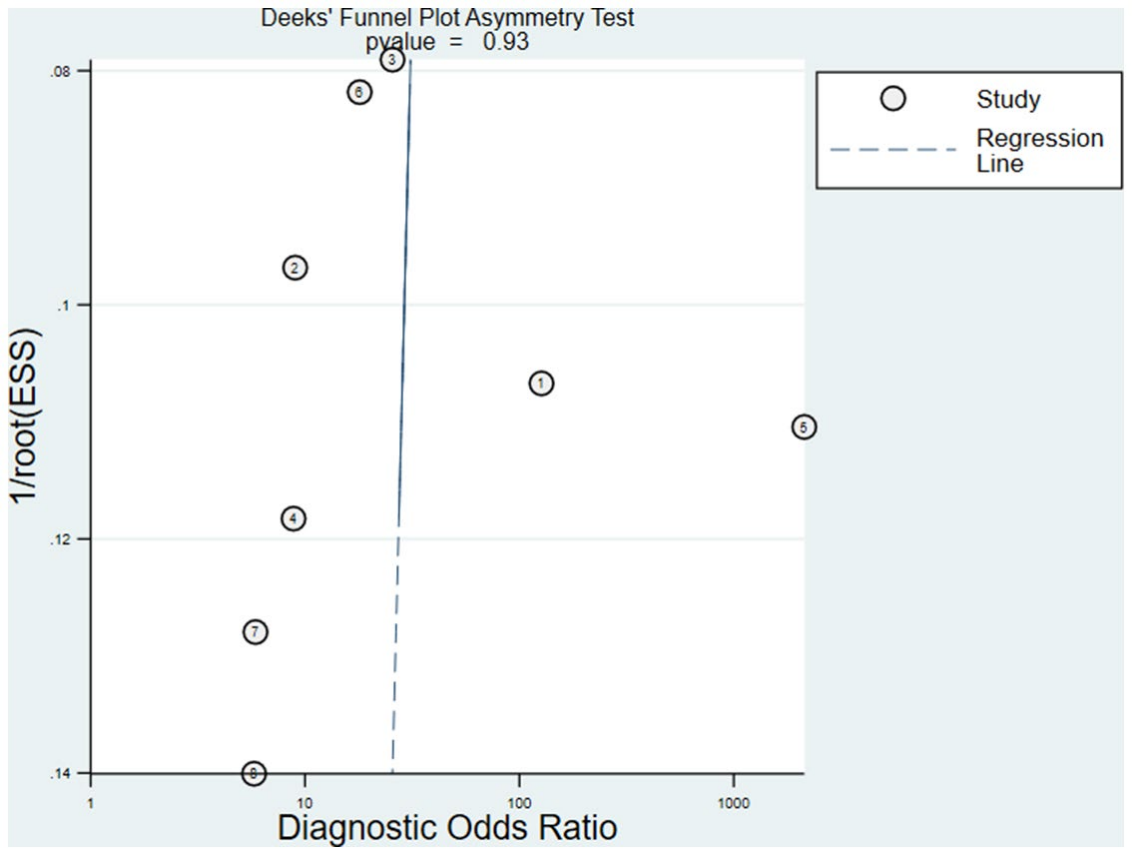
The Deeks’ funnel plot asymmetry test for publication bias showed *p* > 0.05. There was no obvious publication bias in this meta-analysis.

The Deeks’ funnel plot asymmetry test for publication bias showed *p* > 0.05. There was no obvious publication bias in this meta-analysis (Figure 3).

### 3.4. Integrating analysis

**Figure 4:**
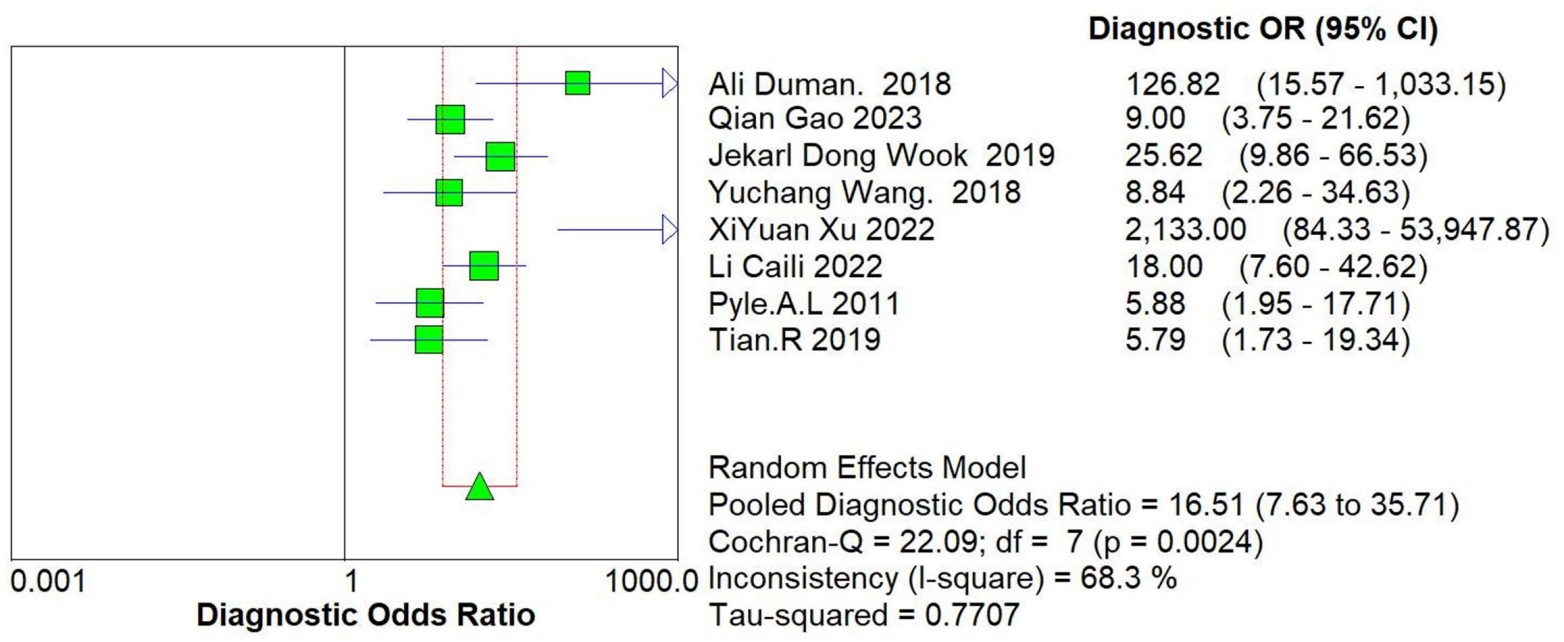
Forest plots of pooled diagnostic OR (95% CI).

The data of this study were imported into the MetaDisc software for analysis. The Spearman correlation coefficient between the sensitivity logarithm and the (1-specificity) logarithm was 0.144 (*p* = 0.734), indicating that the correlation between the two was not significant, which means that there was no threshold effect in this study. The Cochrane-*Q* test of DOR showed that Cochrane-*Q* was 22.09 (*p* < 0.05) and *I*^2^ = 68.3%, indicating there was heterogeneity caused by non-threshold effects in this study. At the same time, this study’s sensitivity, specificity, positive likelihood ratio, and negative likelihood ratio were greater than 50%, so a random effects model was used to combine the above 5 effect sizes (Figure 4).

**Figure 5:**
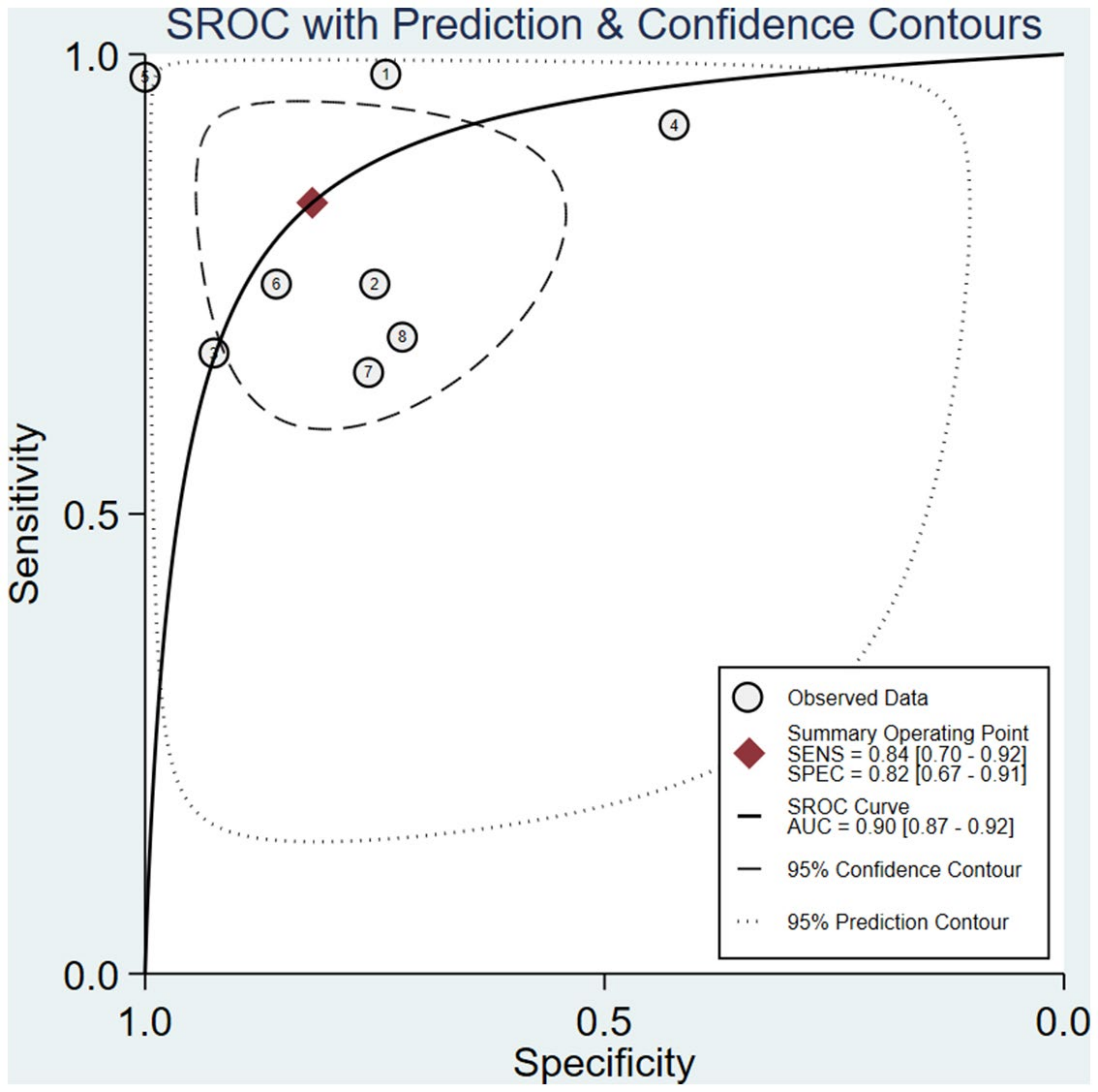
Summary receiver operating characteristic (sROC) curves for eight studies.

**Figure 6:**
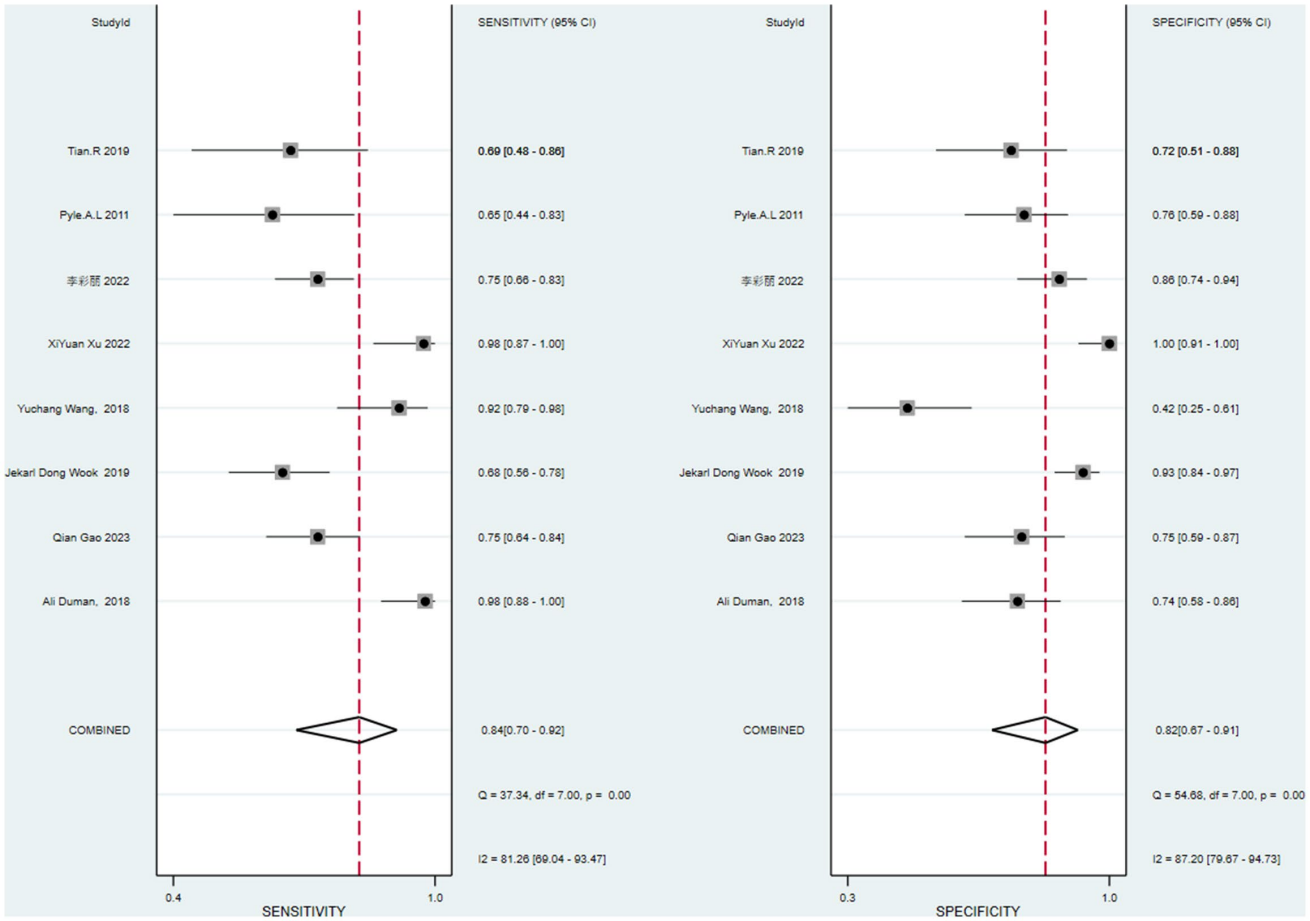
Sensitivity and specificity forest plot of the original study.

**Figure 7:**
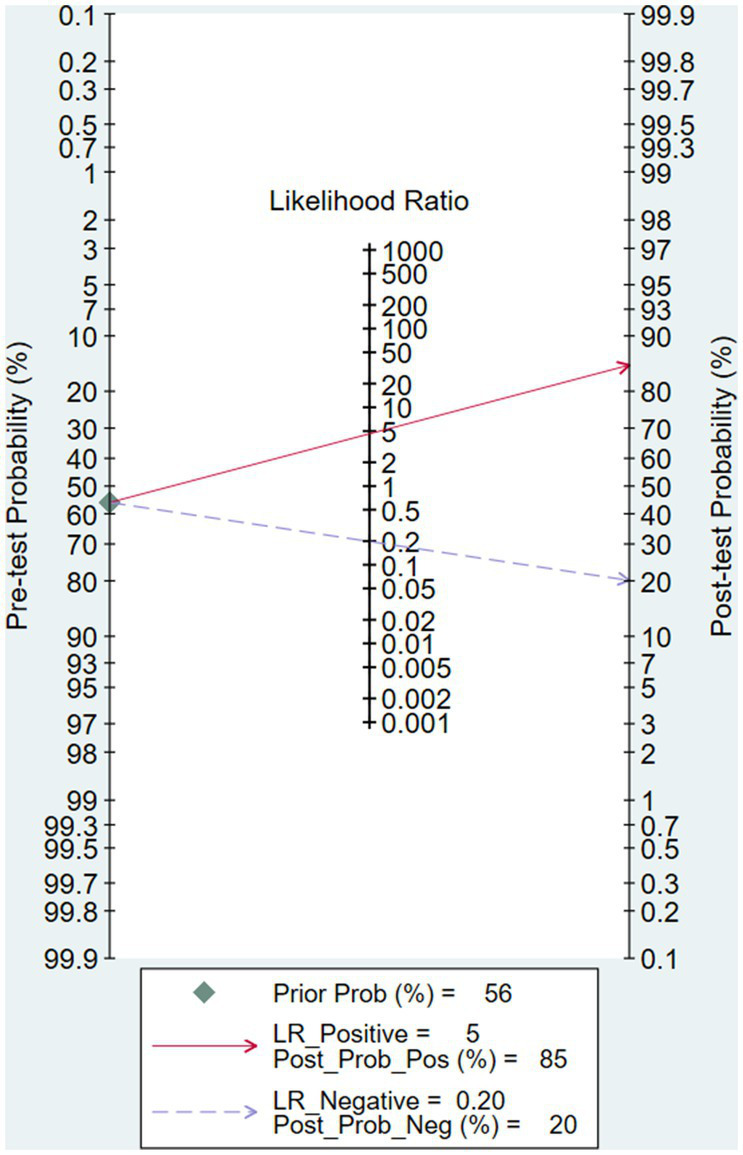
Fagans nomogram showing the posttest probability of sepsis.

After calculation, the combined sensitivity was 0.84 (0.70–0.92), the combined specificity was 0.82 (0.67–0.91) (Figures 5, 6), the combined LR^+^ was 3.711 (2.119–6.500), the combined LR^−^ was 0.287 (0.198–0.415), the combined AUC was 0.90 (0.87–0.92), and the combined diagnostic odds ratio DOR was 16.508 (7.632–35.706). Fagan’s nomogram was used to represent the posterior probability of the pre-test probability of sepsis diagnosis based on the SOFA score (Figure 7). These results confirm the high diagnostic efficiency of MCP-1 in diagnosing sepsis.

### 3.5. Meta-regression and heterogeneous investigations

The study heterogeneity between sensitivity (*I*^2^ = 83.9%) and specificity (*I*^2^ = 87.1%) was higher than the range expected by sampling error. After excluding the heterogeneity caused by the threshold effect, a heterogeneity analysis was conducted on these 8 studies. Single-factor meta-regression analysis was used to explore the source of heterogeneity.

**Figure 8:**
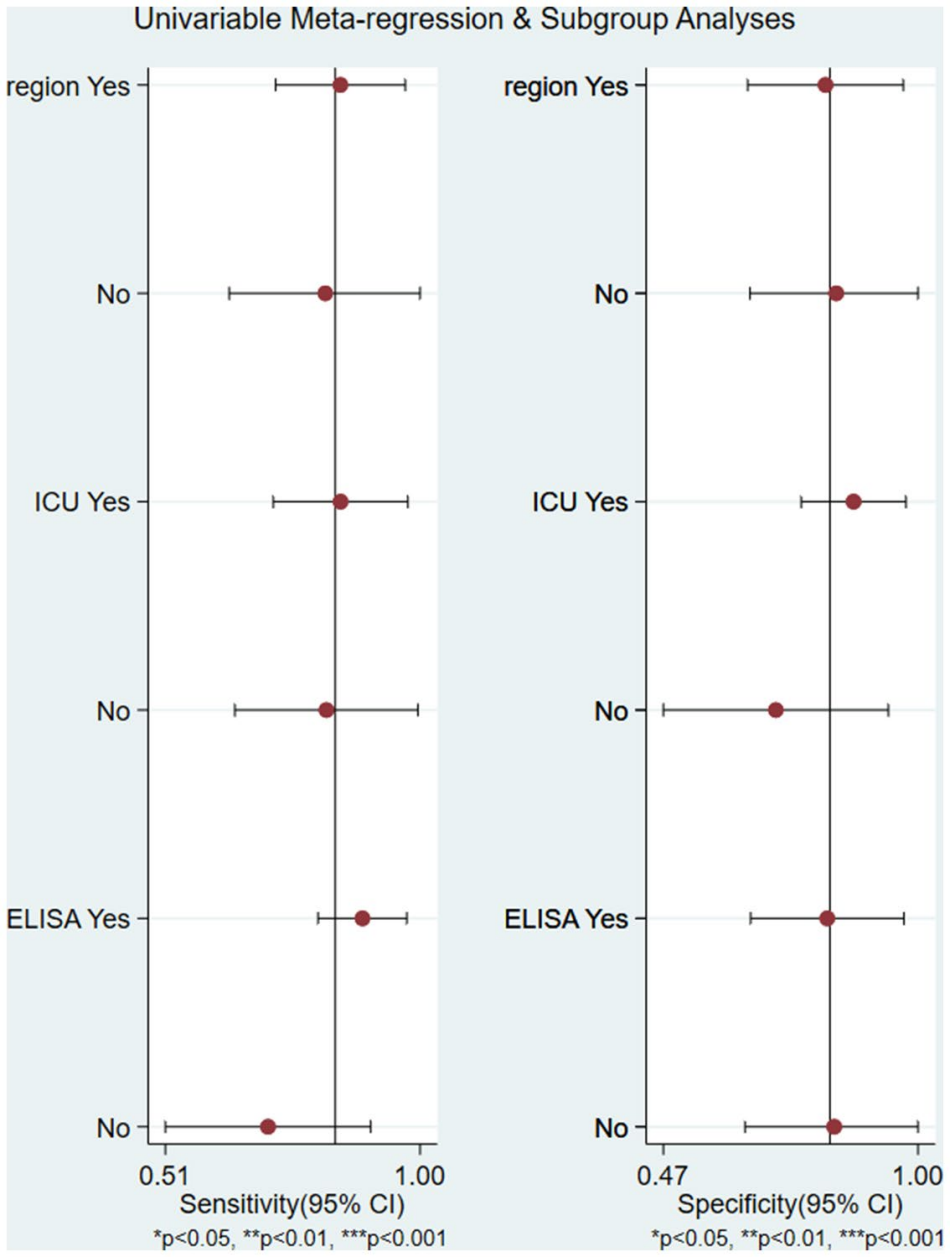
The univariable meta-regression and subgroup analysis (study area, case source, and MCP-1 detection method were covariates) indicated that none of these factors were the source of study heterogeneity (*p* > 0.05) region: China or other country.

The study area, case source, and MCP-1 detection method were covariates. The meta-regression showed that none of these factors were the source of study heterogeneity (*p* > 0.05) (Figure 8). Among all 8 studies, 5 were conducted in the ICU, and 3 were conducted in non-ICU wards. The combined sensitivity and specificity were 79% (95% CI, 73–85) and 85% (95% CI, 80–90) in ICU patients, and 78% (95% CI, 72–83) and 71% (95% CI, 63–79) in non-ICU patients, respectively.

**Figure 9:**
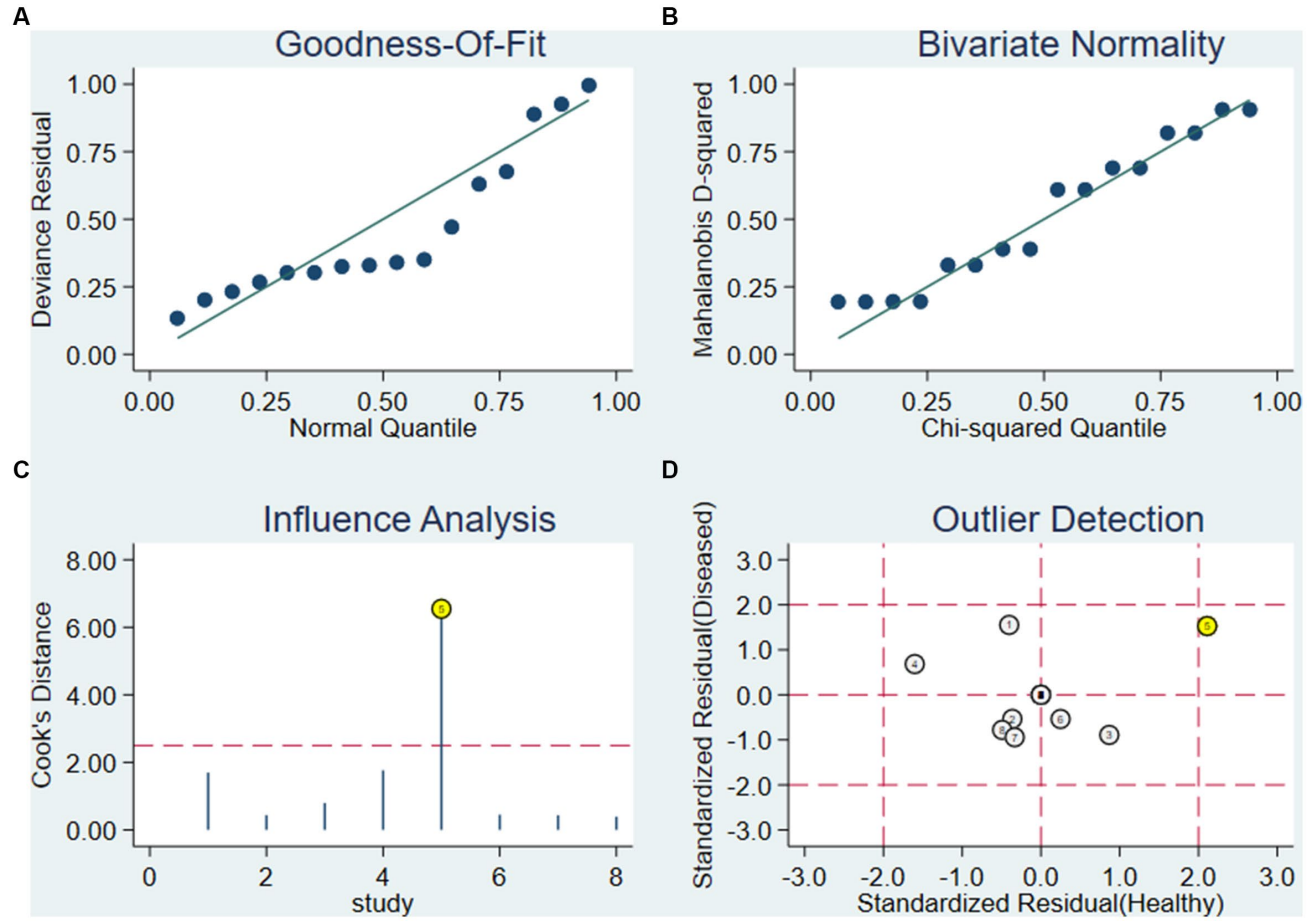
Influence analysis and outlier detection. **(A)** Goodness-of-fit, **(B)** bivariate normality, **(C)** influence analysis and **(D)** outlier detection.

As shown in Figure 9, the goodness of fit and bivariate normality analysis (Figures 9A,B) showed that the model had moderate stability. The influence analysis and outlier detection method (Figures 9C,D) found that 1 original study had strong sensitivity and obvious outliers, while other original studies did not affect the analysis results. Ignoring this study, the combined sensitivity of the other 7 studies decreased to 0.77 (95% CI 0.72–0.81), the combined specificity was 0.78 (95% CI 0.73–0.82), the combined PLR was 3.31 (95% CI 2.03–5.40), the combined NLR was 0.33 (95% CI 0.25–0.43), and the combined DOR was 12.76 (95% CI 7.03–23.17). Overall, the results of this study were relatively stable.

## 4. Discussion

Sepsis is a life-threatening organ dysfunction caused by a dysregulated host response to infection, which was redefined by “Third International Consensus Definition for Sepsis and Septic Shock” guideline in 2016 as an acute endogenous environment with sequential organ failure assessment (SOFA) score greater than or equal to 2 points. The guideline includes laboratory testing recommendations for determining the includes laboratory testing recommendations for determining the sequential organ failure, such as measuring white blood cells (WBCs), platelet counts, bilirubin, and serum creatinine (sCr), to determine the progression of organ dysfunction in sepsis. The results of pathogenic culture are an essential basis for diagnosing sepsis, but due to the long time-consuming, and poor sensitivity, relying on the culture results to diagnose sepsis may delay the condition and even miss the diagnosis. Many studies are looking for ideal biomarkers, but their clinical utility is still being determined. Finding a laboratory index that is easy to obtain, fast, sensitive, and inexpensive to warn against sepsis is possible.

The inflammatory factor MCP-1 mainly induces chemotaxis through the G protein-coupled receptor pathway, regulating the migration and infiltration of monocytes, memory T cells, NK cells, and neutrophils, recruiting them to inflammatory lesions, and participating in the occurrence of inflammation. MCP-1 is also associated with polarized Th2 responses, enhancing T-cell secretion of IL-4 (21). Therefore, this article comprehensively evaluates 8 studies that explored the role of MCP-1 in the early diagnosis of sepsis. The meta-analysis results showed that the area under the sROC curve was 0.90 (0.87–0.92), the diagnostic accuracy of MCP-1 for sepsis was at a moderate level (0.7 ≤ AUC ≤ 0.9) (22). But its wide range of confidence and prediction contours embodies a sense of uncertainty. We analyzed that the different cut-off values of MCP-1 among the included studies, and the large difference between the minimum value and the maximum value, may be one of the factors leading to the wide prediction range and confidence interval of sROC. In addition, the sensitivity analysis showed that Wang’s study may be an influencing factor for such results. Although MCP-1 performed well and AUC was close to 1 in their study, it was not indicated whether the sample was consecutively selected or not, and the samples were from the surgical trauma ward, and the patients with sepsis secondary to trauma surgery were also possible influencing factors. The combined sensitivity was 84%, and the specificity was 82%, both higher than traditional indicators such as PCT (23) and CRP (24).

In recent years, multiple studies have reported that MCP-1 levels can increase in non-infectious diseases such as kidney injury and non-infectious inflammation (25). We speculate that the increased MCP-1 levels in non-septic patients may be a partial reason for the moderate accuracy of MCP-1 in distinguishing septic patients from non-septic patients. Assuming a pre-test probability of 56%, an LRP of 5, and using MCP-1 for sepsis diagnosis would increase the post-test probability to 85%. The LRN was 0.2, and the detection probability decreased to 20% after detecting MCP-1, indicating that MCP-1 testing has a moderate value for sepsis diagnosis. However, a differential diagnostic specificity of 85% or more must be called a highly specific diagnostic test for a definitive diagnosis. Considering that the predictive AUC of PCT in infected patients is 0.85 (21), the overall diagnostic accuracy, sensitivity, and specificity of MCP-1 are superior to traditional inflammatory markers.

So far, no known biomarker as a single test has sufficient (over 90%) sensitivity and specificity to diagnose sepsis and infection (26). Combining several markers seems to be an effective method to improve the accuracy of sepsis diagnosis. Besides MCP-1, ISS (injury severity score), and CD14^+^ CD16^++^ (non-classical) monocyte levels were found to be independent predictors of sepsis, their combination had a better diagnostic effect on sepsis, with AUC areas under the ROC curve of 87% and 79% (15, 19). According to Gao et al. (15), older people’s monocytes secreted more IL-8 and MCP-1 than younger people which may exacerbate the imbalance between inflammatory factors. MCP-1 has the highest predictive value for the onset of inflammatory cytokines (IL-6, IL-8, IL-10, MCP-1) in plasma and also can predict the 30 days mortality of sepsis. In Jekral’s et al. study (16), MCP-1 also showed a higher sensitivity than TNF-α and IL-2. And it did not have an age-dependent plasma level in septic patients (18) showed that plasma MCP-1 levels could distinguish between sepsis and septic shock early.

Furthermore, the meta-regression and sensitivity analysis were conducted to reduce heterogeneity. As a single indicator of diagnostic test performance, DOR is independent of disease prevalence. The range of DOR included in the study was 7.632 to 35.706, with a combined DOR of 16.508. Meta-regression was conducted on the regions, source departments, and detection methods included in the study. The results showed that these 3 factors might not be the primary sources of heterogeneity, and further exploration of the sources of heterogeneity is needed. Sensitivity analysis showed that the study by Xu et al. (20) might also be a source of heterogeneity. The evaluation test results were determined under the knowledge of the gold standard results, and the small sample size of only 80 cases may increase the probability of type II errors.

## 5. Limitation

Our study has some limitations. All included studies selected detection thresholds to optimize sensitivity and specificity, which may lead to an overestimation of test performance. Besides, some clinical diagnoses of sepsis patients lacked microbiological evidence, which may lead to a certain degree of misclassification bias. Further multicenter studies with more patients are needed.

## 6. Conclusion

In summary, MCP-1 has great potential value for the early diagnosis of sepsis, and its role in the development of sepsis can be further studied through large-scale research in the future.

## Data availability statement

The original contributions presented in the study are included in the article/Supplementary material, further inquiries can be directed to the corresponding author.

## Author contributions

ZC contributed to the study design, search strategy, performed the screening, data extraction, and statistical analysis, as well as wrote the manuscript. CL performed the screening, assisted with screening studies and data extraction. JY helped us resolve the conflict and performed a quality assessment. All authors contributed to the article and approved the submitted version.

## Funding

This work was funded by Item Number TYU001EN, Beijing Health Alliance Charitable Foundation.

## Conflict of interest

The authors declare that the research was conducted in the absence of any commercial or financial relationships that could be construed as a potential conflict of interest.

## Publisher’s note

All claims expressed in this article are solely those of the authors and do not necessarily represent those of their affiliated organizations, or those of the publisher, the editors and the reviewers. Any product that may be evaluated in this article, or claim that may be made by its manufacturer, is not guaranteed or endorsed by the publisher.
