# Supplementary material for: Monocyte chemoattractant protein-1 as a potential marker for patients with sepsis: a systematic review and meta-analysis
Source: Front Med (Lausanne). 2023 Sep 1;10:1217784. doi: 10.3389/fmed.2023.1217784 (PMC10502711; doi:10.3389/fmed.2023.1217784)
Supplement: Supplementary file 1 [file Table_1.DOCX]

1. PubMed

((("Sepsis"[Mesh]) OR ((((((((((((((((Bloodstream Infection[Title/Abstract])) OR (Bloodstream Infections[Title/Abstract])) OR (Infection, Bloodstream[Title/Abstract])) OR (Pyemia[Title/Abstract])) OR (Pyemias[Title/Abstract])) OR (Pyohemia[Title/Abstract])) OR (Pyohemias[Title/Abstract])) OR (Pyaemia[Title/Abstract])) OR (Septicemia[Title/Abstract])) OR (Septicemias[Title/Abstract])) OR (Poisoning, Blood[Title/Abstract])) OR (Blood Poisoning[Title/Abstract])) OR (Blood Poisonings[Title/Abstract])) OR (Poisonings, Blood[Title/Abstract])) OR (Severe Sepsis[Title/Abstract])) OR (Sepsis, Severe[Title/Abstract])) AND ((Monocyte chemoattractant factor-1[Title/Abstract]) OR (MCP-1[Title/Abstract]))) AND (sensitiv*[Title/Abstract] OR sensitivity and specificity[MeSH] OR (predictive[Title/Abstract] AND value*[Title/Abstract]) OR predictive value of tests[MeSH] OR accuracy*[Title/Abstract])

1. Cochrane Library

((Bloodstream Infection):ab,ti,kw OR (Bloodstream Infections):ab,ti,kw OR (Infection, Bloodstream):ab,ti,kw OR (Pyemia):ab,ti,kw OR (Pyemias):ab,ti,kw OR (Pyohemia):ab,ti,kw OR (Pyohemias):ab,ti,kw OR (Pyaemia):ab,ti,kw OR (Pyaemias):ab,ti,kw OR (Septicemia):ab,ti,kw OR (Septicemias):ab,ti,kw OR (Poisoning, Blood):ab,ti,kw OR (Blood Poisoning):ab,ti,kw OR (Blood Poisonings):ab,ti,kw OR (Poisonings, Blood):ab,ti,kw OR (Severe Sepsis):ab,ti,kw OR (Sepsis, Severe):ab,ti,kw) AND ((Monocyte chemoattractant factor-1):ab,ti,kw OR (MCP-1):ab,ti,kw) AND ((sensitive):ab,ti,kw OR (sensitivity and specificity):ab,ti,kw OR (predictive):ab,ti,kw OR (predictive value of tests):ab,ti,kw OR (accuracy):ab,ti,kw)

1. Embase

('sepsis':ab,ti OR 'bloodstream infection':ab,ti OR 'bloodstream infections':ab,ti OR 'infection, bloodstream':ab,ti OR 'pyemia':ab,ti OR 'pyemias':ab,ti OR 'pyohemia':ab,ti OR 'pyohemias':ab,ti OR 'pyaemia':ab,ti OR 'pyaemias':ab,ti OR 'septicemia':ab,ti OR 'septicemias':ab,ti OR 'poisoning, blood':ab,ti OR 'blood poisoning':ab,ti OR 'blood poisonings':ab,ti OR 'poisonings, blood':ab,ti OR 'severe sepsis':ab,ti OR 'sepsis, severe':ab,ti) AND ('monocyte chemoattractant factor-1':ab,ti OR 'mcp-1':ab,ti) AND ('sensitive':ab,ti OR 'sensitivity and specificity':ab,ti OR 'predictive':ab,ti OR 'predictive value of tests':ab,ti OR 'accuracy':ab,ti)

1. Web of Science

((ALL=(Bloodstream Infection OR Bloodstream Infections OR Infection, Bloodstream OR Pyemia OR Pyemias OR Pyohemia OR Pyohemias OR Pyaemia OR Pyaemias OR Septicemia OR Septicemias OR Poisoning, Blood OR Blood Poisoning OR Blood Poisonings OR Poisonings, Blood OR Severe Sepsis OR Sepsis, Severe OR Sepsis)) AND ALL=(Monocyte chemoattractant factor-1 OR MCP-1)) AND ALL=(sensitive OR sensitivity and specificity OR predictive OR predictive value of tests OR accuracy)

1. Scopus

( TITLE-ABS-KEY ( "Bloodstream Infection" OR "Bloodstream Infections" OR "Infection, Bloodstream" OR "Pyemia" OR "pyemia" OR "bohemia" OR "bohemians" OR "Pyaemia" OR "pyaemia" OR "Septicemia" OR "septicemia" OR "Poisoning, Blood" OR "Blood Poisoning" OR "Blood Poisonings" OR "Poisonings, Blood" OR "Severe Sepsis" OR "Sepsis, Severe" OR "Sepsis" ) ) AND ( TITLE-ABS-KEY ( "Monocyte chemoattractant factor-1" OR "MCP-1" ) ) AND ( TITLE-ABS-KEY ( "sensitive" OR "sensitivity and specificity" OR "predictive" OR "predictive value off tests" OR "accuracy" ) )

1. CNKI

( ( 主题%='脓毒症' or 题名%='脓毒症' ) AND ( ( 主题%=xls('MCP-1') or 题名%=xls('MCP-1') ) OR ( 主题%='单核细胞趋化因子-1' or 题名%='单核细胞趋化因子' ) ) )

1. CBM

("脓毒症"[常用字段] OR "Sepsis"[常用字段] OR "脓毒血症"[常用字段] OR "脓血症"[常用字段] OR "败血病"[常用字段] OR "严重败血症"[常用字段] OR "脓毒症"[主题词]) AND "MCP-1"[常用字段] AND ("诊断"[常用字段] OR "Diagnosis"[常用字段] OR "诊断和检查"[常用字段] OR "诊断"[主题词])

1. Wanfang Data

((主题=脓毒症) AND (主题=MCP-1)) AND (主题=诊断)

Search Range :From the establishment of the citation library to 02/20/2023.
